# Double-encircling continuous suture technique for complete cyclodialysis: a consecutive case series

**DOI:** 10.1007/s10384-026-01367-8

**Published:** 2026-04-30

**Authors:** Takashi Ueta, Rei Sakata, Megumi Honjo

**Affiliations:** Department of Ophthalmology, Graduate School of Medicine and Faculty of Medicine, The University of Tokyo, 7-3-1, Hongo, Bunkyo, Tokyo 113-8655 Japan

**Keywords:** Cyclodialysis, Cyclopexy, Hypotony, Ciliary body, Surgical procedures

## Abstract

**Purpose:**

To evaluate the safety and efficacy of a novel double-encircling continuous suture technique for the surgical repair of complete (360°) cyclodialysis.

**Study design:**

Retrospective interventional case series.

**Methods:**

Medical records of seven consecutive eyes from seven patients with complete cyclodialysis treated at the University of Tokyo Hospital between 2022 and 2025 were retrospectively reviewed. The extent of cyclodialysis and postoperative anatomical outcomes were assessed using anterior segment optical coherence tomography (OCT). Intraocular pressure (IOP) and best-corrected visual acuity (BCVA) were recorded both pre- and post-operatively. The surgical technique consisted of placing two laps of encircling continuous 10-0 polypropylene sutures at 3–4 mm and 2–3 mm posterior to the limbus. In phakic eyes, phacoemulsification with intraocular lens and capsular tension ring implantation were performed concurrently.

**Results:**

Four men and three women with a mean age of 43.6 ± 25.4 years were included. Cyclodialysis resulted from blunt trauma (n = 3) or surgical complications (n = 4). Preoperative IOP ranged from 2 to 8 mmHg, and hypotonic maculopathy was observed in all cases. Postoperatively, anatomical reattachment of the ciliary body and resolution of hypotony were achieved in six of seven eyes (85.7%). All eyes demonstrated improved BCVA, though the improvement depended on comorbid conditions such as glaucoma, pathologic myopia, and bullous keratopathy. IOP transiently elevated postoperatively, and additional glaucoma surgery was necessary in two cases with a history of glaucoma.

**Conclusion:**

Reproducible safety and efficacy were confirmed for the double-encircling continuous suture technique in repairing complete cyclodialysis, yielding anatomical success and functional improvement.

## Introduction

Cyclodialysis is an uncommon but potentially sight-threatening condition defined as the separation of the ciliary muscle fibers from the sclera, caused by trauma or ocular surgeries [1, 2]. The anomalous communication between the anterior chamber and the suprachoroidal space facilitates enhances outflow through a cyclodialysis cleft and frequently culminates in ocular hypotony. Chronic hypotony, if unresolved, can lead to hypotony maculopathy and impaired visual prognosis [1, 2].

To date, various surgical techniques including internal [3, 4] and external [5–8] cyclopexy, vitrectomy with endotamponade [9, 10], cryotherapy [11], encircling scleral band [12, 13], and internal tamponade using a Cionni-modified capsular tension ring (CTR) [14] are reported to have achieved anatomical closure and intraocular pressure restoration.

We recently developed a novel surgical technique specifically designed for the management of complete (360°) cyclodialysis [15]. The technique, double-encircling continuous suture around the limbus combined with phacoemulsification and CTR insertion, had been performed for intractable cyclodialysis and hypotonic maculopathy, and resulted in successful anatomical and functional recovery [15].

In the present study, we expand upon our preliminary report by describing our experience with seven consecutive eyes in seven patients presenting with complete cyclodialysis. We detail the surgical methodology, evaluate anatomical and visual outcomes, and discuss the theoretical mechanisms that underlie the success of this approach. To our knowledge, this is the largest consecutive case series addressing complete cyclodialysis using a unified, reproducible technique, and it offers new insights into the surgical management of this rare vision-threatening condition.

## Methods

This retrospective study included seven eyes of seven patients with complete cyclodialysis, all treated at the Department of Ophthalmology, The University of Tokyo Hospital, between 2022–2025. Institutional review board (IRB) approval was obtained (#2217), and all procedures conformed to the tenets of the Declaration of Helsinki.

Medical records were reviewed to extract demographic and clinical data, including the underlying etiology, intraocular pressure (IOP), and best-corrected visual acuity (BCVA) measured with a Landolt C chart and converted to logMAR for this study. The diagnosis and circumferential extent of the cyclodialysis (i.e., ciliary body-scleral separation) and its cleft (i.e., visible communication between the anterior chamber and the suprachoroidal space) were determined preoperatively using anterior segment optical coherence tomography (AS-OCT; CASIA 2, Tomey Corporation). The presence and resolution of hypotonic maculopathy were assessed using spectral-domain OCT (SD-OCT; Spectralis, Heidelberg Engineering) of the posterior pole. Postoperative anatomical outcomes, including the re-apposition of the ciliary body to the sclera, were confirmed using AS-OCT.

**Fig. 1:**
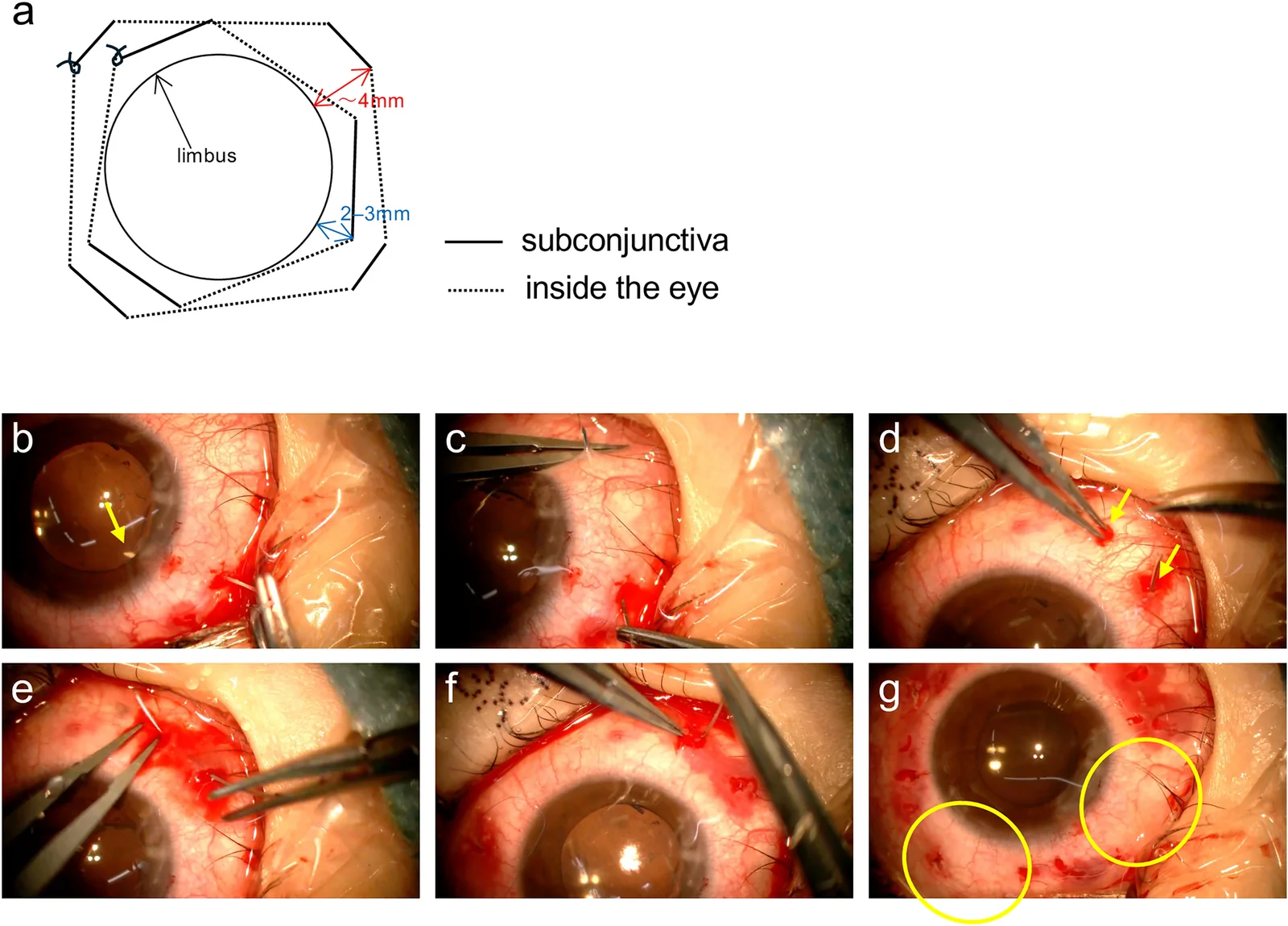
Schematic drawing illustrating the surgical technique. and solid and dashed lines are 10-0 polypropylene running through the subconjunctiva and inside the eye, respectively (**a**). Surgical images present the visual confirmation of the needle piercing through the whole thickness of the ciliary body (arrow) (**b**), the needle comes in and out of the eye (**c**), small conjunctival incisions at the locations where the needle pass through the sclera (arrow) (**d**), the needle passing through the subconjunctival space (**e**), the needle again coming into the eye (**f**). After the suturing procedure, some area of the conjunctiva can be maintained intact (circled area) (**g**)

All surgery was performed by one of the authors (T. U.). All patients underwent surgical repair using a novel double-encircling continuous suture technique designed to address both the circumferential and radial separation of the ciliary body (Fig. 1). Before suturing, the looped structure of the 10-0 polypropylene monofilament (PC-9, Alcon Surgical) was released by cutting one thread at the base of the needle. Two continuous encircling sutures using PC-9 were passed around the limbus at 3–4 mm and 2–3 mm posterior to the limbus. The sutures were placed with care to ensure full-thickness transfixion of the ciliary body, which was confirmed intraoperatively by direct visualization of the needle tip through the dilated pupil. When tying the suture, care was taken to avoid excessive tightening so as to prevent deformation of the globe. Each suture knot was buried within a partial-thickness scleral incision to prevent exposure. In the initial two cases, limbal peritomy was performed to facilitate suture placement as previously described [15]. In the subsequent five cases, only localized conjunctival incisions were made to minimize conjunctival damage (Fig. 1). Because of the length and flexibility of the PC-9 needle, suturing at 2–3 mm posterior to the limbus carries a risk of inadvertent contact with the crystalline lens. Therefore, phacoemulsification (PEA) with a three-piece intraocular lens (IOL) implantation was performed preemptively in all phakic eyes. In addition, a capsular tension ring (CTR, CTR130P, HOYA Surgical Optics) was implanted to minimize postoperative capsular contraction, which could otherwise exert tractional force on the ciliary body and compromise its reattachment to the sclera.

Postoperative follow-up included serial measurements of IOP and BCVA, AS-OCT imaging to confirm anatomical reattachment of the ciliary body, and SD-OCT evaluation of the macula. Data were presented as mean ± standard deviation.

## Results

Consecutive seven cases of complete cyclodialysis treated with our technique were identified since 2022. Characteristic and surgical results of each case are summarized in Table 1. The mean age of the patients was 43.6 ± 25.4 years old, four men and three women. Cyclodialysis occurred after blunt trauma in three cases and as a surgical complication in the other four cases. The detail of the previous intraocular surgery before the development of cyclodialysis varied; pars plana vitrectomy (PPV)+PEA+IOL for macular hole in other hospital (case #3), PPV+scleral fixation of IOL for a dislocated IOL plus a history of trabeculectomy five years earlier (case #4), Two surgical procedures of trabeculotomy *ab interno* (case #5), cataract surgery complicated with posterior capsule rupture, CTR insertion, and dropped IOL in another hospital, referred to our department with bullous keratopathy, dropped IOL, and hypotonic maculopathy due to cyclodialysis (case #6).

**Table 1.** Characteristics of each case in this study

Case #	Age, yo	Sex	Reasons of cyclodialysis	Previous surgeries for cyclodialysis	Duration of cyclodialysis	Pre-op logMAR	Pre-op IOP, mmHg	Cleft size	Post-op logMAR	Post-op Max IOP, mmHg	Post-op Final IOP, mmHg	Follow-up	Surgery of our technique
1	19	M	Blunt trauma (baseball)	2 cyclopexies	7 mo	1.3	2	180°	0	38 (POD3)	12	10 mo	Cyclopexy+PEA+IOL+CTR
2	53	F	Bicycle accident	none	6 mo	1.5	8	180°	0.2	52 (POD1)	13 on 3 IOP-lowering drops	7 mo	Cyclopexy+PEA+IOL+CTR
3	76	F	Surgery (PPV+PEA+IOL)	Cyclopexy+Cryo+gas	20 mo	1.0	2	None	0.8^a^	2	2	8 mo	Cyclopexy
4	70	M	Surgery (PPV+IOL scleral fix)	none	2 w	HM	0	30°	1^b^	25 (POD7)	10 after needling at POD14	8 mo	Cyclopexy+1.3ml 100% SF6
5	20	F	2^nd^ trabeculotomy *ab interno*	Cyclopexy	8 mo	1.2	1	120°	0.5^c^	59 (POD3)	10 after PFM (POD11) and needling (POD28), and on 2 IOP-lowering drops	5 mo	Cyclopexy+PEA+IOL+CTR
6	52	M	Surgery (PPV, failure of CTR insertion, failure of IOL scleral fix)	none	10 mo	HM	4	300°	1.4^d^	41 (POD2)	13	4 mo	Cyclopexy+12.5%C3F8 exchange
7	15	M	Blunt trauma (soccer)	Cyclopexy	4 mo	1.1	4	120°	0	42 (POD2)	13	2 mo	Cyclopexy+PEA+IOL+CTR

*PPV* pars plana vitrectomy, *PEA* phacoemulsification, *IOL* intraocular lens, *CTR* capsular tension ring, *IOP* intraocular pressure, *POD* postoperative day

^a^Visual recovery was not achieved due to persistent hypotonic maculopathy

^b^Visual recovery was limited due to pathologic myopia with choroidal neovascularization

^c^Visual recovery was limited due to glaucoma-associated visual field defect

^d^Visual recovery was limited due to bullous keratopathy

**Fig. 2:**
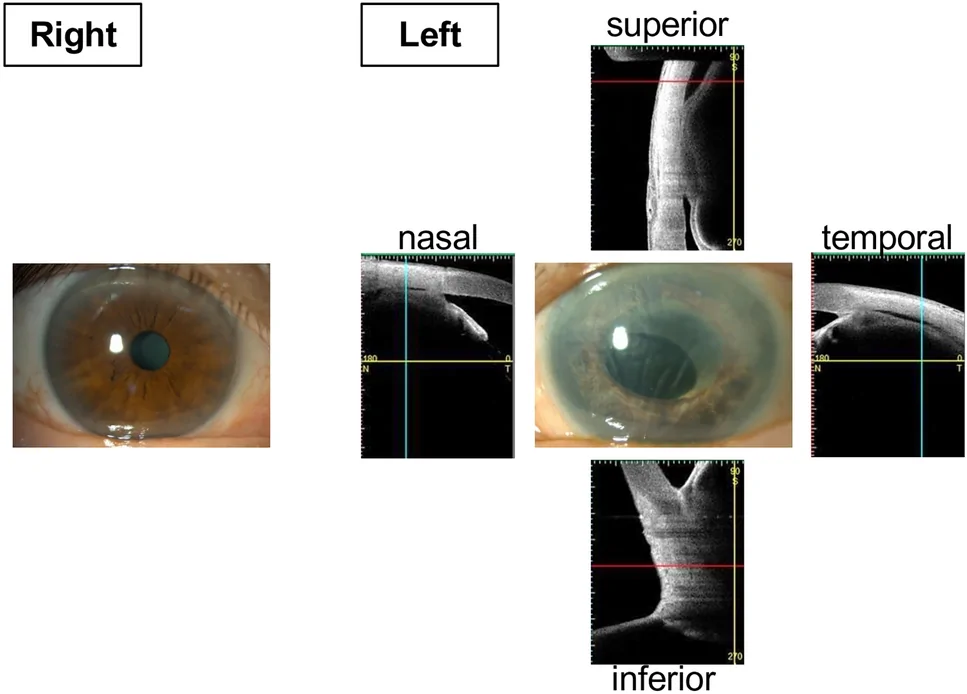
Anterior segment color photographs for both eyes and AS-OCT images for the left eye of case 3 who had undergone surgeries for the cyclodialysis (cyclopexy + cryotherapy + gas tamponade). On initial presentation to our department, decreased corneal endothelial cells and atrophic iris with depigmentation were observed, presumably caused by extensive cryotherapy on the anterior sclera. Complete cyclodialysis was observed, while the cyclodialysis cleft was not identified

Preoperative AS-OCT showed complete cyclodialysis in all cases. On the other hand, the size of the cyclodialysis cleft varied, ranging 1 – 10 clock hours. The cyclodialysis cleft was not identified for case #3 who had already undergone surgical treatment (cyclopexy + cryotherapy + gas tamponade) in another hospital. On initial presentation to our department, decreased corneal endothelial cells and depigmented iris presumably caused by cryotherapy on the anterior sclera were noted (Fig. 2); it was not clear whether the hypotony was due to cyclodialysis, impaired ciliary function, or both.

Preoperative IOPs were below 5 mmHg in six cases, and 8mmHg in case #2. Hypotonic maculopathy was evident in all the seven cases, and preoperative BCVA was generally low (range; hand motion–1.0 logMAR).

The surgical treatment always involved cyclopexy of our technique. Additionally, the insertion of CTR and IOL (case #1, #2, #5 and #7), SF6 gas injection (case #4), and C3F8 gas exchange (case #6) were also performed simultaneously.

**Fig. 3:**
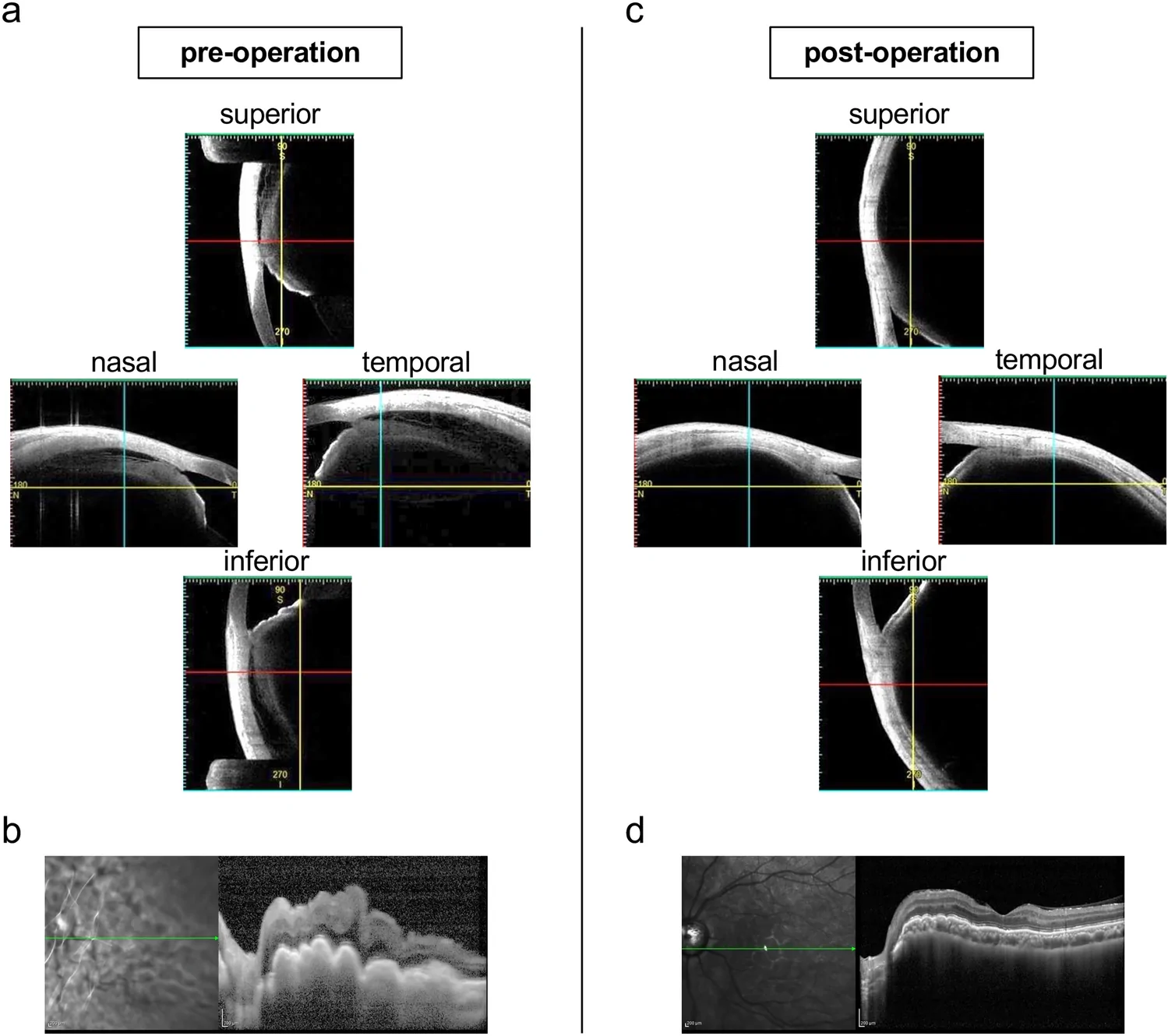
Pre- and post-operative AS-OCT (**a** and **c**) and SD-OCT (**b** and **d**). Pre-operatively, Cyclodialysis was observed in all preoperative AS-OCT images, and cyclodialysis cleft was visualized in the nasal image (**a**). Severe hypotonic maculopathy (**b**). Post-operatively, cyclodialysis was resolved in all AS-OCT images, and improvement of hypotonic maculopathy was also confirmed

Postoperatively, cyclodialysis and hypotonic maculopathy was resolved in six cases (85.7%). Representative AS-OCT images pre- and post-surgery are presented in Fig. 3. In the successful six cases, IOP surged during the first week of post-operation and then gradually decreased to the normal range (Table 1). Except for the increases in IOP, there was no surgery-related complication, and neither vitreous hemorrhage nor retinal detachment did not occur after cyclopexy. In glaucoma eyes (case #4 and #5), additional treatments were necessary, comprising bleb needling (case #4 and #5) and filtration surgery (case #5). Although Case #2 was not originally an eye with glaucoma, three kinds of IOP-lowering drops were still necessary even after seven months of surgery.

Postoperative BCVA was improved in all cases, though it was variable (range; 1.4–0.0) and affected by co-morbidities; pathologic myopia (case #3), glaucoma (case #4 and #5), and bullous keratopathy (case #6).

## Discussions

This case series presents consecutive cohorts of patients with complete (360°) cyclodialysis managed using our novel double-encircling continuous suture technique. The results demonstrate that this method achieves high success rates of anatomical closure and resolution of hypotony, with corresponding improvement in visual acuity, in a condition that is both rare and challenging to manage.

### Comparison with previously reported surgical approaches

Suturing techniques of cyclopexy have been categorized into direct external, indirect external, and indirect approaches. Direct external cyclopexy is suturing the ciliary body to the sclera under direct observation of the cilia after peritomy, partial thickness scleral flap formation, and further small scleral incision to visualize the external surface of the cilia [5, 6]. Indirect external cyclopexy is transscleral suturing of the cilia with or without peritomy [7, 8]. In internal cyclopexy, following lensectomy and vitrectomy, a thread is inserted into the eye from the corneal incision, approaches the internal surface of the cilia, and suturs the cilia to the sclera [3, 4]. Our technique should be categorized as indirect external approach because the cilia is not exposed for direct visualization. Our technique addresses both circumferential and radial dimensions of the detachment by providing uniform appositional force to the ciliary body along the full limbal circumference. The double-encircling configuration at 3–4 mm and 2–3 mm posterior to the limbus reduces the risk of incomplete closure. We consider that the advantages of our technique are (1) it requires a reduced number of stitches ever for complete cyclodialysis with a extensive cyclodialysis cleft; four stiches for the outer circular suture and three for the inner suture; (2) the needle tip that had pierced the ciliary can be observed through the dilated pupil, providing confirmation that the full thickness of the cilia is penetrated by the needle; (3) vitrectomy is not necessary; and (4) a large area of the conjunctiva can be preserved, which could be especially important for glaucomatous eyes.

Surgical strategies other than cyclopexy include vitrectomy with air/gas/silicone oil endotamponade [9, 10], cryotherapy [11], encircling scleral band [12, 13] and internal tamponade using Cionni-modified CTR to the ciliary sulcus [14]. Vitrectotmy is not often desirable for young patients without posterior vitreous detachment. In our case series we used gas tamponade as an adjuvant therapy in two cases of previously vitrectomized eyes. Regarding cryotherapy, extensive treatment may lead to severe damage in the anterior segment of the eye, like in case #3. Encircling scleral band is reported useful through a speculated mechanism of obstructed uveoscleral outflow from the cyclodialysis [12, 13]. Suture of Cionni CTR to the ciliary sulcus may be useful for relatively small cyclodialysis clefts.

### Anatomical considerations and imaging correlation

Interestingly, although all cases in this series were diagnosed with complete 360° cyclodialysis by AS-OCT, the extent of the cyclodialysis cleft evaluated on AS-OCT varied markedly (1–10 clock hours). In our case series, five cases out of seven had cyclodialysis cleft of ≧4 clock hours, indicating that the technique presented in this study may be effective even for severer cases.

The integration of AS-OCT in our workflow allowed objective preoperative mapping of cyclodialysis and postoperative confirmation of reattachment. The imaging modality is especially valuable in extensive cyclodialysis, where gonioscopic visualization can be challenging due to hypotony. Furthermore, because cyclodialysis cleft was not visible in case #3, AS-OCT may be helpful in differentiating hypotony caused primarily by cyclodialysis from that due to other reasons including ciliary malfunction.

### Postoperative IOP dynamics and clinical implications

In the postoperative IOP we observed a transient early surge followed by stabilization, consistent with the previous studies [3, 5, 7, 10, 11, 14, 15]. The surge likely reflects acute cessation of abnormal uveoscleral outflow, with subsequent normalization as aqueous production and trabecular outflow equilibrate. In eyes with coexisting glaucoma or prior filtering surgery (Cases #4 and #5), this normalization process was complicated by residual filtration pathways, necessitating additional interventions such as bleb needling or repeated filtration surgery. The persistence of elevated IOP in Case #2 despite the absence of glaucoma history suggests that postoperative IOP control may be influenced by other factors, including ciliary body hyperfunction after prolonged hypotony, steroid response, or subclinical angle changes. These findings underscore the importance of anticipatory postoperative IOP management, particularly in eyes with glaucoma, prior filtration procedures, or advanced optic nerve damage. Proactive planning for early IOP spikes with close monitoring may reduce the risk of optic nerve injury during the postoperative period.

### Limitations

This study has several limitations. First, the retrospective design and small sample size limit the generalizability of our findings. However, the rarity of complete 360° cyclodialysis makes large prospective trials challenging. Second, the follow-up period, though adequate for assessing anatomical closure and early functional outcomes, may not fully capture long-term prognosis. Third, variability in concomitant procedures (e.g., CTR/IOL implantation, gas tamponade) introduces heterogeneity that could influence both anatomical and functional outcomes. Finally, the lack of a direct comparison group prevents formal evaluation of superiority over alternative surgical methods.

## Conclusion

Our experience demonstrates that the double-encircling continuous suture technique provides an effective, reproducible method for repairing complete cyclodialysis, with high rates of anatomical success, resolution of hypotony, and meaningful visual improvement. The technique’s design addresses both circumferential and radial detachment dimensions and is adaptable to eyes with complex surgical histories. While further validation in larger cohorts is needed, this approach offers a promising surgical option for one of the most challenging entities in anterior segment and ciliary body pathology.
